# Role of medical resource level in iodine deficiency disorder

**DOI:** 10.1186/s40779-017-0126-5

**Published:** 2017-06-01

**Authors:** Chen Xu, Zhen Liang, Yong-Jun Luo

**Affiliations:** 1 Department of Military Medical Geography, College of High Altitude Military Medicine, Third Military Medical University, Chongqing, 400038 China; 2 Battalion 5 of Cadet Brigade, Third Military Medical University, Chongqing, 400038 China; 3 Key Laboratory of High Altitude Medicine (PLA), Third Military Medical University, Chongqing, 400038 China

**Keywords:** Iodine deficiency disorders (IDDs), Health level, Medical security, China

## Abstract

**Background:**

Iodine deficiency disorders (IDDs) refer to a series of diseases caused by the human body's insufficient iodine intake. Edible salt became iodized in China in 1996, which yielded remarkable results. We have known that IDDs is associated with iodine in the human body, but it is not clear whether IDDs is related to medical resource level.

**Methods:**

We collected the number of IDDs cases and an index for the level of medical resource from 31 provinces, autonomous regions and municipalities directly under the central government in China. All data came from the China Statistical Yearbook of Health and Family Planning issued in 2013 by the Peking Union Medical College Publishing House. Data standardization and linear regression analysis were used.

**Results:**

The results showed that IDDs correlated with the number of beds in medical and health institutions, number of medical health personnel, number of medical and health institutions, total health expenditure, average health expenditure per capita, medical insurance for urban resident and new rural cooperative medical rural residents (*P* < 0.01). In a multiple linear regression, IDDs was most significantly associated with the number of beds in hospitals, the number of rural health personnel, the number of basic medical and health institutions and government health expenditure for these institutions.

**Conclusion:**

Based on the experimental data, we concluded that IDDs had a positive connection with the medical resource level, and basic and rural areas had a more significant association with IDDs. This analysis provides new and explicit ideas for iodine prevention and control work in China.

## Background

Iodine deficiency disorders (IDDs) is the most widely distributed endemic disease and end angers the largest number of people in the world. One-third of the world’s population lives in iodine-deficient areas, and 32 countries have a low level of iodine intake [1, 2]. In the 1970s, iodine deficiency disorders could be observed at different epidemic levels in China, with the threatened population amounting to approximately 370 million [3]; 35 million endemic goiter patients and 250 thousand endemic cretinism patients were observed [4, 5].

Iodine is an indispensable microelement of the human body and plays a key role in metabolism, including enhancing protein synthesis, promoting growth and maintaining normal brain function [6]. Additionally, iodine is an essential element for normal thyroid function. The common symptoms of IDDs are endemic goiter, cretinism, abortion, stillbirths, congenital malformationand intellectual disturbance [7–9]. IDDs is primarily observed in regions away from the coasts and at high altitudes, such as mountainous areas where the soil, water and food sources contain less iodine. In addition, genetic factors cannot be ignored [10], but prenatal diagnosis is limited and has not spread to some rural areas in China. In addition, due to a lack of knowledge regarding IDDs, people often think that it is not a genetic disease, and IDDs is often ignored in prenatal diagnosis, making it difficult to prevent IDDs based on this factor.

In view of the most important problem, in 1995, the Chinese government started to implement a universal salt iodization (USI) campaign [11], which achieved a significant effect. According to the national IDDs surveillance data, household coverage rates with iodized salt increased from 39.9 to 95.3% [12]. As a result, by the end of 2010, 28 provinces in China (regions and cities) had achieved the provincial target of eliminating iodine deficiency disorder, and 97.9% of counties (regions and cities) met the goal of eliminating iodine deficiency disorder. Tibet, Xinjiang, and Qinghai completed the targets for the current stage to eliminate iodine deficiency diseases. The latest adjustment of iodine content in salt occurred in March 2012 and finished in March 2014. To prevent the population from the potential side effects caused by excessive iodine intake, the new national standard for salt iodization have been narrowed from 20 to 50 mg/kg to 18–39 mg/kg, which both lowers the iodine content in iodized salt and narrows down the range of iodine content allowed [13]. Over the past 20 years, China and other countries have conducted thorough studies on the prevention and treatment of IDDs, adopting USI as the main approach and supplementing it by strengthening publicity and health education measures [14]. By some measures, China has already achieved great success, but IDDs is not completely under control. Further, the amount of iodine intake has a U-type curve relationship with thyroid disease [15, 16]; excessive intake of iodine would cause a high incidence of iodine goiters, iodine-induced hyperthyroidism [17, 18], and hypothyroidism [19], which could potentially develop into autoimmune thyroid disease (AITD) [20] and thyroid cancer [21, 22]. After implementation of iodized salt, cities near the deluged Yellow River appeared to have high levels of iodine originating from a water source, which presented an increased incidence of these diseases.

In previous studies, environmental and genetic factors were identified as the most important factors for IDDs but could not be quantified. To further improve IDDs prevention, treatment plans and continuous elimination and to achieve the ultimate goal of entirely eliminating IDDs, not only should we research iodine in a scientific manner, but we should also find other related factors for these diseases. We wanted to research the secondary factors and discover what were the most correlated with IDDs. As we all know, the local medical resource level determines the popularity rate of USI and the early diagnosis of the disease. We usually diagnose goiter by touch and visual inspection. Many years ago, WHO and the International Council classified IDDs into grades 0, 1, and 2; because of the limitations of diagnosis, levels 0 and 1 of IDDs were usually missed, which was contrary to the aim of early diagnosis and treatment of IDDs. Now, with improvement in the level of medical resources, B-ultrasounds and fine-needle aspiration (FNA) are used for the early diagnosis of IDDs [23]. In addition, awareness of IDDs has increased gradually, which can improve the rate at which people visit clinics; a better level of medical resource can improve the detection rate of IDDs. Therefore, we speculate that the reason for not obtaining effective control is potentially correlated with the medical resource level. Because such a study has not been conducted to date, we wanted to explore this relationship. Thus, we selected seven indicators that could satisfactorily describe the medical resource level of health facilities (the number of beds in medical and health institutions), medical health personnel, medical and health institutions, total health expenditure, average health expenditure per capita, medical insurance for urban residents and new rural cooperative medical rural residents. Then, we conducted a mathematical statistical analysis and explored the relationship between IDDs and level of medical resource.

## Methods

### Data collection

We collected the number of IDDs cases (IDDs cases primarily included endemic goiter, endemic cretinism and endemic subclinical cretinism);in summary, we collected a total of 5,126,941 patients with IDDs, which included 4,800,287 patients with goiter, 224,834 patients with two degrees of goiter, and 101,820 patients with cretinism. The number of beds in medical and health institutions, the number of medical health personnel, the number of medical and health institutions, total health expenditure, the average health expenditure per capita (not including Tibet in the abovementioned two indexes), medical insurance for urban residents, new rural cooperative medical rural residents (not including Tianjin) and other relevant information were collected from 31 provinces, autonomous regions and municipalities directly under the central government (not including Hongkong, Macao and Taiwan) in China in 2013. All data came from the China Statistical Yearbook of Health and Family Planning issued in 2013 by the Peking Union Medical College Publishing House. The data in the China Statistical Yearbook of Health and Family Planning issued in 2013 are excerpted for annual health statistics; a part of data came from sample surveys; data on the population and community economy came from the China Statistical Yearbook issued in 2013 by China Statistics Press; statistical information came from the Ministry of Public Security, Ministry of Education and Ministry of Civil Affairs; and medical insurance data for urban residents came from the Ministry of Human Resources and Social Security in China.

### Statistical analyses

Using Microsoft Office Excel 2007 software, we conducted a data analysis of the respective correlation between IDDs cases and the seven indicators and their concrete indexes (Table 1). To guarantee the reliability of the results and eliminate the effects from the data among different units and quantity levels, we adopted min-max data normalization and drew a line chart that reflected the degree of correlation to intuitively represent the degree relevance [24].

**Table 1:** Seven indicators and their concrete indexes that influenced the local level of medical resource

Item		Concrete indexes
Number of beds in medical and health institutions (A)	a1	Beds in hospitals
	a2	Beds in basic medical and health institutions
	a3	Beds in professional medical and health institutions
	a4	Beds in other medical and health institutions
Medical health personnel (B)	b1	City medical personnel
	b2	Rural medical personnel
Medical and health institutions (C)	c1	Hospitals
	c2	Basic medical and health institutions
	c3	Professional medical and health institutions
	c4	Other medical and health institutions
Total health expenditure (D, hundred million yuan)	d1	Government expenditure on health
	d2	Social health expenditure
The average health expenditure per capita (E, yuan) -		
Medical insurance for urban residents (F) -		
New rural cooperative medical rural residents (G) -		

We used SPSS 13.0 software to conduct a regression analysis of the above data. Regression analysis is a statistical analysis technique that we use to confirm the quantitative relation of which two or more variables depend. After the result was determined, we analyzed the value of significance (sig.), which was the significance coefficient of the regression relation and the actual significance probability of the value of *F*, namely, the value of *P*. We considered *P* < 0.05 to be statistically significant. The numbers of IDDs cases were defined as dependent variables, and the seven indicators were defined as independent variables. First, we used linear regression to analyze the relationship between the number of IDDs cases and the seven indicators. Second, there were four concrete indexes for the number of beds in medical and health institutions,two concrete indexes for the number of medical health personnel, four concrete indexes for the number of medical and health institutions and two concrete indexes for total health expenditure; we can see these in the support table. A multiple regression was preformed: the number of IDDs cases were defined as the dependent variable, and for total health expenditure, government expenditure on health and social health expenditure were the concrete indexes defined as independent variables. We used the same method to analyze the other three indicators. We adopted a multiple linear regression equation and specifically determined which concrete indexes had the greatest impact on IDDs by comparing the value of the standardized regression coefficient Beta with sig. The standardized regression coefficient (Beta value) was used to compare the importance among variables in multiple regressions [25]. When the items that needed to be compared had the same measurement unit, a larger Beta value (standardized coefficient) indicated that the item would have a greater impact on the variables. This study was approved by the ethical committee of the Third Military Medical University in China.

## Results

**Fig. 1:**
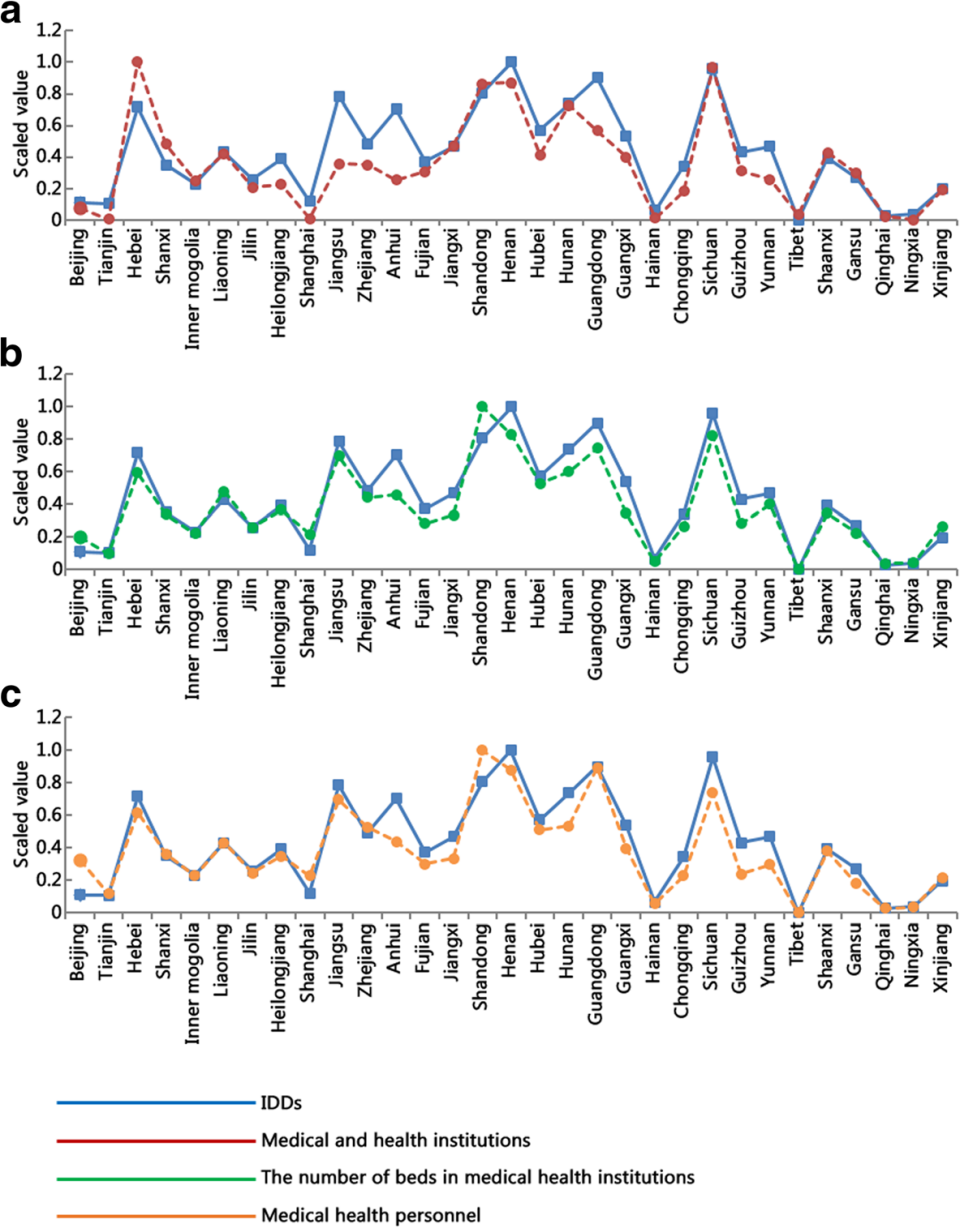
Correlations of IDDs with the number of medical and health institutions (**a**), beds in medical and health institutions (**b**), and medical health personnel (**c**)

**Fig. 2:**
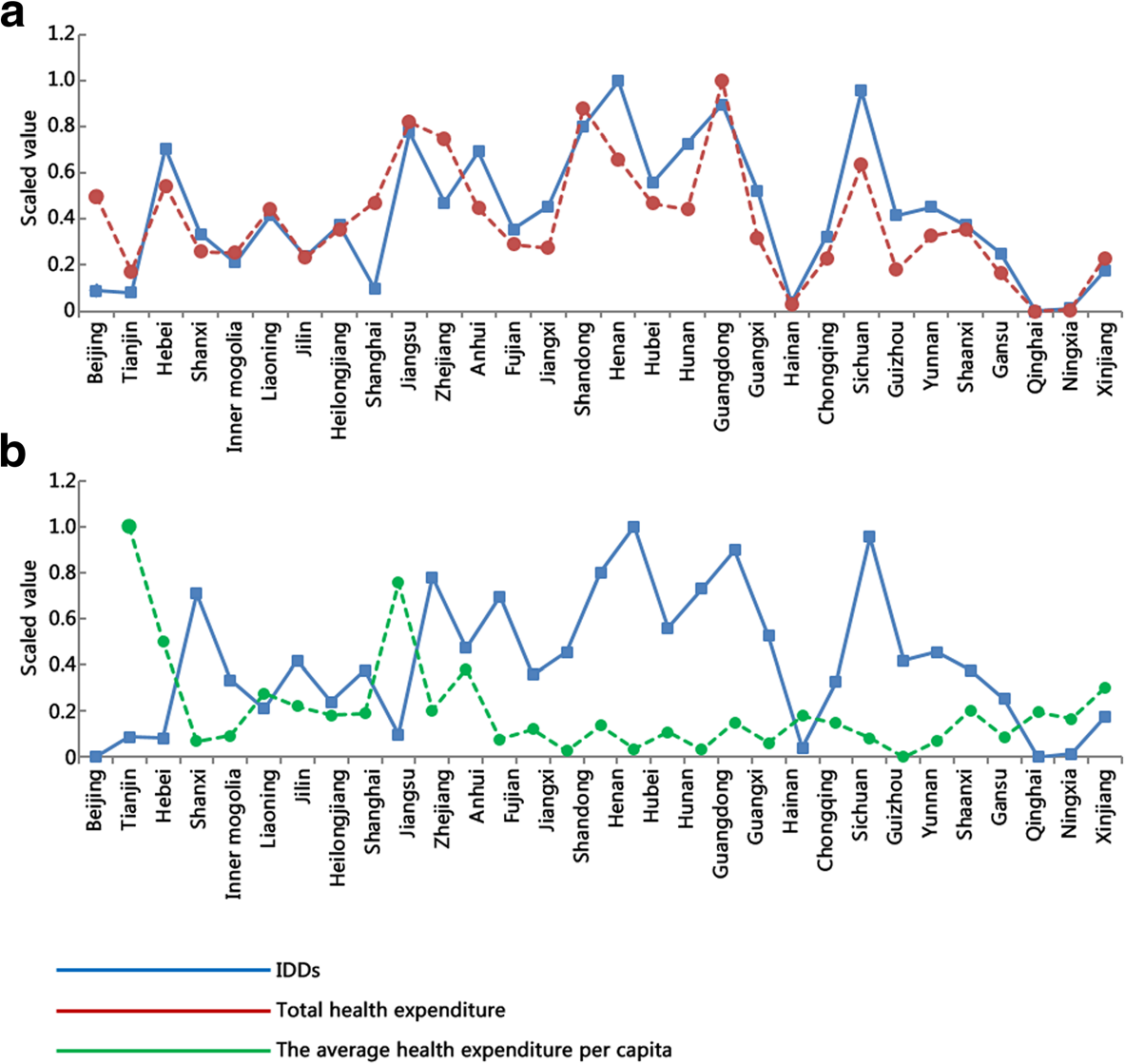
Correlations of IDDs with health expenditure. The line charts show the total health expenditure (**a**) and the average health expenditure per capita (**b**)

**Fig. 3:**
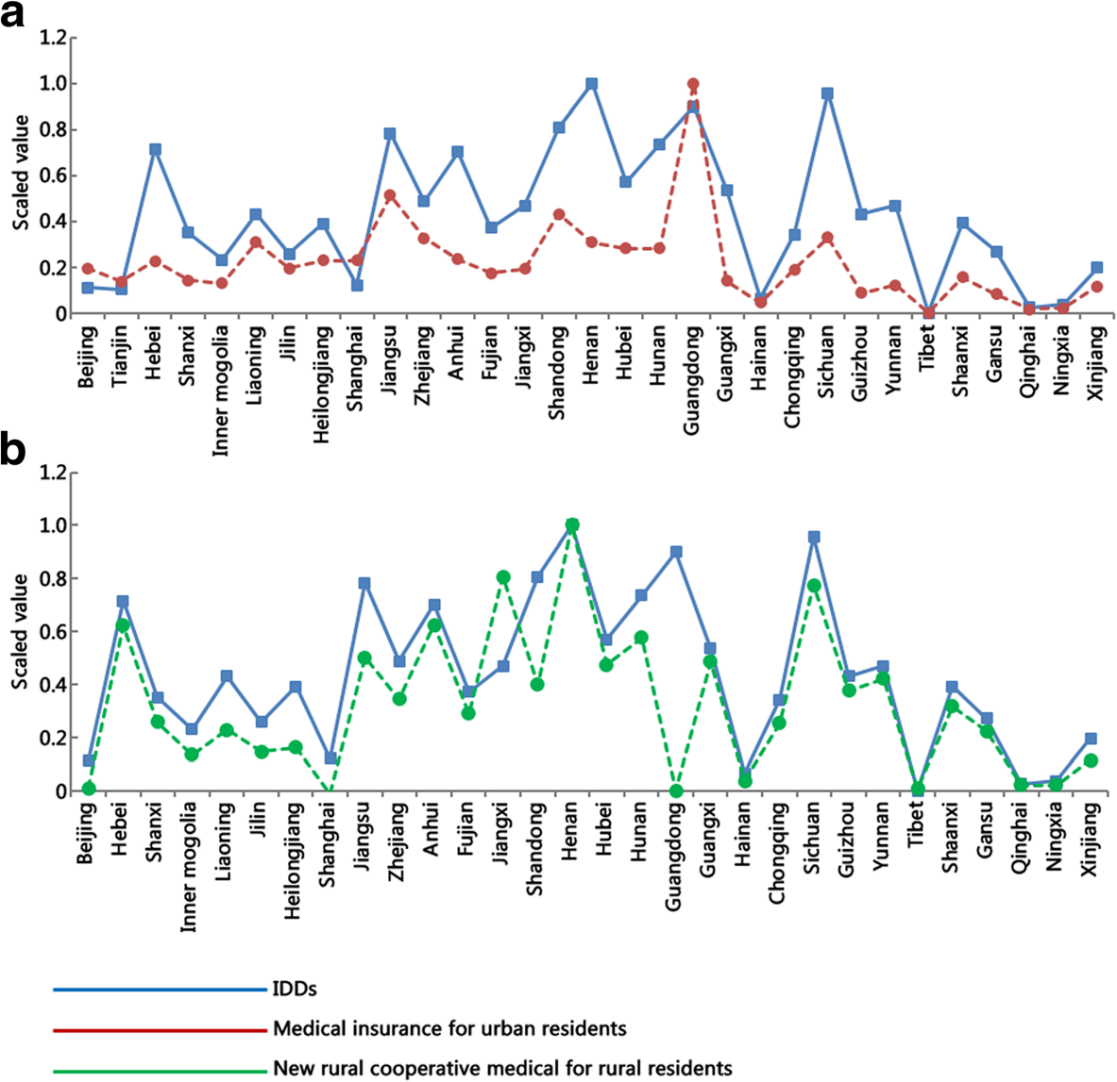
Correlations of IDDs with medical security. The line charts show medical insurance for urban residents (**a**) and new rural cooperative medical rural residents (**b**)

The data were standardized and are shown in line graphs (Figs. 1, 2 and 3). We can directly see the correlations between the number of IDDs cases and possible influencing factors. Except for the average health expenditure per capita, all factors showed correlations with IDDs. In addition, should we draw conclusions based on mathematical statistics, we can determine that the average median urinary iodine concentration of children aged 8–10 years is 205.58 μg/L in 20 provinces, and Tianjin, Shanxi, Inner mogolia, Jilin, Heilongjiang, Jiangsu, Hunan, Chongqing, Guizhou, Gansu, Qinghai provinces did not provide the data about the average medium urinary iodine concentration.

Correlations between the number of IDDs cases and possible influencing factors are shown in Table 2. The linear regressions revealed significant associations between IDDs and the number of beds in medical and health institutions (standardized coefficient: 0.948, *P* < 0.001), the number of medical health personnel (standardized coefficient: 0.926, *P* < 0.001), the number of medical and health institutions (standardized coefficient: 0.868, *P* < 0.001), total health expenditure (standardized coefficient: 0.785, *P* < 0.001), the average health expenditure per capita (standardized coefficient: −4.87, *P* < 0.01), medical insurance for urban residents (standardized coefficient: 0.694, *P* < 0.001), and new rural cooperative medical for rural residents (standardized coefficient: 0.768, *P* < 0.001). All groups exhibited statistical significance. Correlations between the number of IDDs cases and the concrete indexes are shown in Table 2 (except for the number of beds in other medical and health institutions, standardized coefficient: 0.314, *P* = 0.086). In Table 3, a multivariate regression analysis revealed that the number of beds in hospitals (standardized coefficient: 0.453, *P* = 0.002) and the number of beds in basic medical and health institutions (standardized coefficient: 0.401, *P* = 0.003) were statistically significant; the number of beds in professional medical and health institutions (standardized coefficient: 0.148, *P* = 0.176) was not significantly associated with IDDs. City medical personnel and rural health personnel had a remarkable correlation with IDDs (standardized coefficient: 0.260, *P* = 0.002; standardized coefficient: 0.773, *P* = 0.000, respectively). There was only one related factor: basic medical and health institutions were statistically and significantly correlated with IDDs (standardized coefficient:0.454, *P* = 0.004); hospitals, professional medical and health institutions and other medical and health institutions were not significantly associated with IDDs. Government expenditure on health (standardized coefficient: 1.044, *P* = 0.000), social health expenditure (standardized coefficient: −0.600, *P* = 0.000), and personal health expenditure (standardized coefficient: 0.374, *P* = 0.016) were all statistically and significantly correlated with IDDs.

**Table 2:** Association of influencing factors with IDDs

Variable	B	Std. Error	Beta	*t*	*P* (Sig.)
The number of beds in medical and health institutions	0.021	0.001	0.948	16.070	0.000
The number of medical health personnel	0.013	0.001	0.926	13.188	0.000
The number of medical and health institutions	0.107	0.011	0.868	9.418	0.000
Total health expenditure (hundred million yuan)	4.600	0.687	0.785	6.698	0.000
Average health expenditure per capita (yuan)	−1.604	0.544	−4.87	−2.951	0.006
Medical insurance for urban residents	1.460	0.281	0.694	5.192	0.000
New rural cooperative medical for rural residents	0.965	0.152	0.768	6.343	0.000

**Table 3:** Multiple linear regression analysis results

Variable	B	Std.Error	Beta	*t*	*P* (Sig.)
The number of beds in hospitals	0.015	0.004	0.453	3.455	0.002
The number of beds in basic medical and health institutions	0.032	0.010	0.401	3.290	0.003
The number of beds in professional medical and health institutions	0.071	0.051	0.148	1.390	0.176
The number of city medical personnel	0.008	0.002	0.260	3.401	0.002
The number of rural health personnel	0.017	0.002	0.773	10.133	0.000
The number of hospitals	1.202	1.193	0.190	1.007	0.323
The number of basic medical and health institutions	0.057	0.018	0.454	3.185	0.004
Professional medical and health institutions	3.012	2.380	0.221	1.265	0.217
Other medical and health institutions	7.489	5.086	0.159	1.478	0.151
Government expenditure on health (hundred million yuan)	23.385	3.197	1.044	7.316	0.000
Social health expenditure (hundred million yuan)	−8.432	1.349	−0.600	−6.249	0.000
Personal health expenditure (hundred million yuan)	5.825	2.250	0.374	2.590	0.016

## Discussion

In previous studies, researchers focused more on genetic and environmental factors, believing that those were the main factors causing IDDs. Additionally, researchers focused on the extremely low content of iodine in the soil, various chemical compounds in water [26], insufficient iodine intake of the human body [27], the distribution of drinking water [28], gender, age, tobacco smoking, and alcohol, which could also cause the risk factors of IDDs [10]. Based on these studies, the WHO has clearly shown the successful implementation of preventive measures for IDDs, vitamin A deficiency in children, IDDs in pregnant women or infants, etc. China’s government also took appropriate measures to prevent IDDs, but the disease has not yet been completely controlled. However, few studies have explored the association between IDDs and the medical care level. This study evaluated this relation; we analyzed the data and got results that exceeded our expectations. It was easy for us to discover that the number of IDDs cases had a positive correlation with the number of beds in medical and health institutions, number of medical health personnel, number of medical and health institutions, total health expenditure, average health expenditure per capita, medical insurance for urban residents and new rural cooperative medical rural residents (*P* < 0.01).

We attempted to analyze the fundamental reasons for why higher levels of medical resource were associated with greater numbers of IDDs cases. First, local people had a high level of health care awareness and paid comparatively more attention to prevention and control of the disease when the level of medical resource was higher, which enabled publicity efforts regarding the disease to go more smoothly. As people gradually developed IDDs, they would go to hospitals as long as there were clinical manifestations. Thus, with the clinic rate increasing, the number of cases of IDDs will increase, which comprehensively and accurately provides data about this epidemic situation. These data are critical for the prevention and treatment of IDDs in the next step of the plan. Second, a high level of medical resources represents more advanced detection techniques and a higher overall quality of doctors than in low-level medical resource areas, which are also convenient for diagnosing the disease; Reinhardt et al. [29] also reported different detection methods could affect the diagnosis of IDDs. In fact, in more remote rural areas in China, doctors lacking professional knowledge of IDDs always use touching to diagnose instead of using B-ultrasounds or a comprehensive evaluation; as such, levels 0 and 1 of IDDs were usually missed. An ambiguous diagnosis could not always reflect the actual number of IDDs. Third, we could not ignore the fact that population bases are usually larger in the higher levels of medical resource, and this might be a pivotal factor for why the higher levels of medical resource were associated with the greater number of IDDs cases.

After further multiple linear regression analyses, it could be observed that primary-level medical and health institutions, the number of the rural health personnel, the number of beds in healthcare institutions, and the health expenditure of the government had the highest degree of correlation with the number of IDDs cases. In addition, the number of IDDs cases in rural areas represents a larger proportion, and these results were consistent with the actual situation in China. In fact, there were many difficulties in prevention and treatment of IDDs in rural areas in China. First, affected by traditional habits and economic conditions, the extension of the USI was not sufficient in outlying and poverty-stricken areas [30] and some rural residents refuse to eat refined packaged iodized salt, preferring local traditional rough salt because of the impact on traditions and economic limitations [31]. Second, limited by health expenditure of the local government, IDDs is deficient in propaganda; underestimation of the disease was the biggest problem: people did not know that IDDs could affect the development of intelligence [32]. Third, it was vital to keep a professional team for prevention and control, but we found rural areas lacked health personnel: on average, there were only one health personnel per rural hospital, and their wages were low, they lacked medical technology, and not all factors were conducive to propaganda of IDDs. Fourth, though prenatal screening could diagnose IDDs, in some rural areas limited by inadequate inspection of equipment and technical levels, some high-level iodine areas did not even screen IDDs in prenatal screening. In summary, if the goal of eliminating IDDs is to be pursued further, we must start at the primary level and in rural areas, increase the government's capital investment in health and improve the medical resource conditions to comprehensively promote rural healthcare level.

Conclusively, our study demonstrated that the IDDs diagnosis was closely correlated with the medical level in China, as a larger number of IDDs cases were present in some provinces with a high level of medical resources; we conjecture that this phenomenon is associated with a low visiting rate, a difference in population bases and so on. Governments should pay more attention to the provinces with a low level of medical resources, increasing government expenditure on health and attaching importance to propaganda in order to get comprehensive and accurate data about an epidemic situation; these data are critical for the prevention and treatment of IDDs in the next step of the plan. On the other hand, prevention and treatment of IDDs in rural areas is momentous; improving the level of medical resources in rural areas and enhancing local residents’ awareness of IDDs is essential.

There were some limitations to this experiment. First, the data were not complete: we lacked data about total health expenditure in Tibet and new rural cooperative medical rural residents in Tianjin. Complete data would be preferable in the future to accurately evaluate the influence of the medical and health levels further. Second, the data are cross-sectional and not experimental, which lacks persuasive ability, so the results and conclusions should be further confirmed. Additionally, it would be more accurate to collect the data from each city for the linear regression. A large sample size would be more persuasive.

## Conclusions

Based on the experimental data, we concluded that IDDs had a positive correlation with the medical resource level, and basic and rural areas had a more significant association with IDDs. This analysis provides new and explicit ideas for iodine prevention and control work in China.

## Abbreviations

AITD: Autoimmune thyroid disease
FNA: Fine-needle aspiration
IDDs: Iodine deficiency disorders
USI: Universal salt iodization
